# Validation of Quantitative Magnetic Resonance Cholangiopancreatography Metrics in Prediction of Transplant-free Survival in Primary Sclerosing Cholangitis

**DOI:** 10.1016/j.jceh.2025.103417

**Published:** 2025-11-21

**Authors:** Tim E. Middelburg, Bregje Mol, Carlos Ferreira, Tom Davis, Karin Horsthuis, Ynte S. de Boer, Adriaan J. van der Meer, Annemarie C. de Vries, Roy S. Dwarkasing, Johannes A. Bogaards, Aart J. Nederveen, Jaap Stoker, Cyriel Y. Ponsioen

**Affiliations:** ∗ Department of Gastroenterology & Hepatology, Amsterdam UMC, the Netherlands; † Amsterdam Gastroenterology, Endocrinology & Metabolism, Amsterdam, the Netherlands; ‡ Perspectum Ltd., Oxford, United Kingdom; § Department of Radiology & Nuclear Medicine, Amsterdam UMC, University of Amsterdam, the Netherlands; ¦ Department of Gastroenterology & Hepatology, Erasmus MC, Rotterdam, the Netherlands; ¶ Department of Radiology & Nuclear Medicine, Erasmus MC, Rotterdam, the Netherlands; # Department of Epidemiology & Data Science, Amsterdam UMC, the Netherlands

**Keywords:** biomarker, quantification, prognostication

## Abstract

**Background and aims:**

Qualitative magnetic resonance cholangiopancreatography (MRCP) scoring models in primary sclerosing cholangitis (PSC) are hampered by interobserver variability and evidence for quantitative MRCP has so far been limited by cohort sizes, follow-up time and lack of validation. This study aimed to validate the prognostic value of quantitative MRCP metrics in PSC in a large multicentre cohort.

**Method:**

Retrospective, cross-sectional, clinical and quantified MRCP data by MRCP+ were collected from a non-transplant and transplant centre and randomised (1:1 ratio) into a derivation and validation set. Transplant-free survival, a composite of liver transplantation and PSC-related mortality (excluding colorectal carcinoma), was the primary endpoint. Least absolute shrinkage and selection operator analysis with manual guidance was used to compose a risk classifier. Prognostic performance and risk stratification were expressed by C-statistic and hazard ratios (HRs), and were validated in the validation set.

**Results:**

A total of 224 patients were included with a median 6.8 years (Q1,Q3: 4.5, 9.8) of follow-up from MRCP onwards. Analysis identified number of strictures, proportion of 3–5 mm diameter ducts, years from diagnosis to MRCP and centre type as prognostic. The derived risk classifier showed a C-statistic of 0.72 (95% confidence interval [CI]: 0.60–0.81) and stratified effectively, with high-risk patients having threefold higher HR than low-risk patients (HR, 3.2; 95% CI: 1.6–6.4; *P* = 0.001) in the validation set.

**Conclusion:**

This study confirms the prognostic value of quantitative MRCP (number of strictures and proportion of 3–5 mm diameter ducts) on long-term transplant-free survival in PSC and warrants further study on incorporating quantitative MRCP metrics into existing prognostic risk models.

## Graphical abstract

**Figure:**
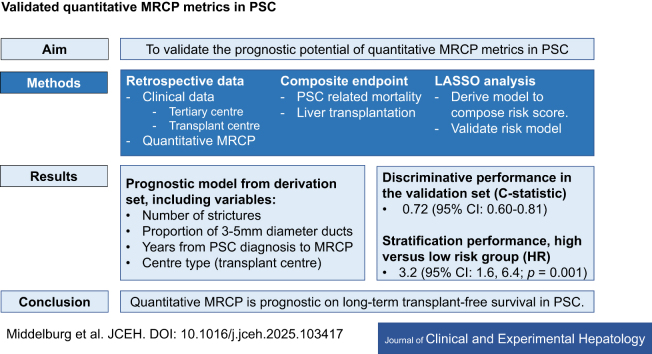

Primary sclerosing cholangitis (PSC) is a rare, chronic cholestatic liver disease that is characterised by intrahepatic and extrahepatic strictures and dilatations of the biliary tree. The associated progressive process of liver fibrosis leads to end-stage liver disease. Currently, there is no treatment available to halt disease progression and liver transplantation (LT) is deemed the only curative option despite a 25% recurrence rate after transplantation.^1^

Monitoring progression is important for counselling and to detect complications of PSC in an early stage to improve survival.^2^ Prognostic models such as the Amsterdam–Oxford model (AOM), partly based on biochemical markers, were developed to aid prognostication.^3^, ^4^, ^5^, ^6^ However, as current biochemical markers can fluctuate or may only be elevated in end-stage liver disease, the long-term prognostic performance of these models is suboptimal. Imaging-based scores were developed because of their presumed face validity nature. Rajaram *et al.* modified the endoscopic retrograde cholangiopancreatography (ERCP)-based classification of Majoie and demonstrated the correlation with estimated survival.^7^^,^^8^ Tenca *et al.* refined and tested this scoring system on magnetic resonance cholangiopancreatography (MRCP), preferred because of the noninvasive nature, and found moderate agreement between both modalities.^9^ Two qualitative scores by Ruiz *et al.*, based on morphological changes, portal hypertension and intrahepatic biliary dilatation in non-contrast scans, and morphological changes with parenchymal enhancement heterogeneity in gadolinium-enhanced scans, showed a significant association with future radiological progression.^10^ Grigoriadis *et al.* introduced the semiquantitative biliary assessment (DiStrict score) that showed promising prognostication of transplant-free survival.^11^ Unfortunately, interobserver variability and the lack of external validation of most scores hamper current clinical use.

Quantitative analysis of the biliary tree might overcome these limitations. Several publications on MRCP+ (Perspectum Ltd., Oxford), a post-processing software that utilises artificial intelligence (AI) to generate quantitative metrics from MRCP images, demonstrated the prognostic potential of quantitative MRCP. Previously identified prognostic MRCP+ metrics in PSC are proportion of bile ducts with a diameter of 3–5 mm,^12^^,^^13^ the number of biliary strictures^14^, ^15^, ^16^ and median duct diameter as associated with event-free survival.^13^ However, a recent consensus statement by the European Society for Gastrointestinal and Abdominal Radiology (ESGAR) still emphasised the need for more robust prove as current studies are limited by relatively small cohorts, short to moderate follow-up time, homogenous population and the lack of cross-centre validation.^17^

Hence, we aimed to validate the prognostic potential of MRCP+ metrics in a large heterogenous population-based Dutch prospective cohort with long follow-up.

## METHODS

### Study Design

This retrospective, cross-sectional study utilised a prospective cohort including PSC patients participating in the EpiPSC2 study, a nation-wide Dutch population-based prospective registry with approval of an institutional review board. For this study, data from the Amsterdam University Medical Center (UMC; tertiary centre) and Erasmus MC (transplantation centre) were used. Data were collected from December 2004 up to February 2025 and pseudonymized. Given the retrospective design, a formal study size was not calculated. This study was conducted in agreement with the ethical guidelines of the Declaration of Helsinki. Written informed consent was given by active participants of the EpiPSC2 study.

### Selection

Inclusion criteria were as follows: patients of 18 years or older with large-duct or small-duct PSC diagnosis according to the international PSC study group (IPSCSG) guidelines.^18^ Available 1.5 T or 3T 3D-MRCP were selected. Patients with features of autoimmune hepatitis (AIH), as defined according to the simplified AIH criteria, were not excluded.^19^ PSC patients with liver transplantation or extensive biliary surgery, except for cholecystectomy, prior to 3D-MRCP acquisition were excluded. A minimal follow-up of 1 year after 3D-MRCP acquisition with successful MRCP+ analysis without transplantation or PSC-related death was required for inclusion. Concurrent cholestatic diseases, viral hepatitis and abusive alcohol use were not observed in this cohort.

### Magnetic Resonance Imaging Scans

Both 1.5T and 3T magnetic resonance imaging scans were included from a local archive if at least one 3-dimensional (3D) MRCP was available. To match MRCP+ acquisition protocol requirements, scans had to have been acquired with multishot fast/turbo spin on Siemens, Philips or GE vendors. Furthermore, parameter requirements included a range of 40–240 continuous slices with a 1.0–2.0 mm slice thickness and pixel resolution varied between 0.43 × 0.43 and 1.56 × 1.56 with a maximum 2:1:1 voxel ratio. As archival MRCP data were used, details regarding scanning protocols are not available. It is expected that patients were routinely fasting for at least 4 h prior to the MRCP and scans from 2017 onwards were performed according to protocols as recommended by the IPSCSG in 2017.^20^ Images were pseudonymized according to local privacy protocols.

### MRCP+ Analysis

**Figure 1:**
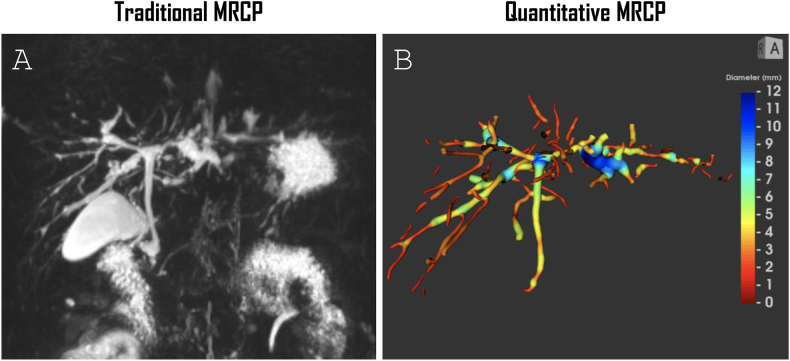
**MRCP and MRCP+ of a 28-year-old male PSC patient.** (A) Maximum intensity projection image (MIP) of a 3D-MRCP of the biliary tree from a 28-year-old male PSC patient and a corresponding (B) MRCP+ 3D-model with colour coding by duct diameter, excluding the gallbladder and pancreatic duct. PSC: primary sclerosing cholangitis; 3D: three-dimensional; MRCP: magnetic resonance cholangiopancreatography.

MRCP+ is a software tool that generates an estimated volumetric model of the biliary tree as shown in Figure 1a–d, that allows an extensive quantitative assessment including ducts diameter, the number of ducts and number of strictures (narrowing when compared to adjacent ducts by more than 1 mm in absolute terms and more than 30% in relative terms) among others, further described extensively by Goldfinger *et al.* and Cristoferi *et al.*^14^^,^^21^ Pseudonymized 3D-MRCP images were uploaded to a secure portal to allow post-processing analysis by MRCP+™. Pre-analysis was performed to evaluate the parameters described in the previous chapter and eligible MRCP images were assessed by operators on breathing artefacts, motion artefacts, lack of biliary contrast or gastrointestinal contamination to produce a reliable quantitative model. Previous studies have shown the reproducibility and repeatability of MRCP+.^16^^,^^21^ Based on experience with MRCP+ in previous publications and internal consistency tests, the included MRCP+ metrics were limited to number of ducts, the sum of dilated duct length, number of dilated ducts, proportion of ducts with a median diameter of 3–5 mm, the sum of stricture length, total number of strictures and number of ducts with a stricture of dilatation.

### Clinical Data Collection

Data were collected from the EpiPSC2 registry and included: sex, date of birth, date of PSC diagnosis, type of PSC, features of AIH, use of ursodeoxycholic acid, cholecystectomy and indication, type of inflammatory bowel disease (IBD), closest laboratory analysis to MRCP date (± 3 months). For type of IBD, ulcerative colitis (UC) was used as reference versus absence or Crohn’s disease according to the risk factors for PSC described by Weissmuller *et al.*^22^ Data regarding LT, indication of LT, death and the reason of death were collected. AOM risk scores were determined for patients with available data and missing data were not imputed.

### Statistical Analysis

Participants from both centres were included in one cohort to be divided via seeding, by a 1:1 ratio, into a derivation and validation cohort. Categorical data are displayed as numbers (percentages) and continuous data as mean (standard deviation) or median (interquartile ranges, Q1-Q3). For evaluation of centre differences, Kruskal–Wallis analysis was used for numerical data and Chi-squared test (>5 expected counts) or Fisher’s exact test (<5 expected counts) for categorical data.

The primary endpoint, referred to as transplant-free survival, was a composite including LT- or PSC-related death, excluding death from colorectal carcinoma (CRC). The composite of LT- and PSC-related death including death from CRC and a composite of death by all causes or LT were used as secondary endpoints in sensitivity analysis. Because previous publications showed high correlations between MRCP+ metrics, Pearson’s correlation analysis was performed.^23^ For the derivation of predictive capacity of MRCP+ metrics, the derivation set was used for metric selection by least absolute shrinkage and selection operator (LASSO) analysis. LASSO was preferred over traditional Cox stepwise regression because of expected correlated MRCP+ metrics and the high number of covariates in relation to the number of events.^24^, ^25^, ^26^ The coefficient generated by LASSO, indicating an increase in log hazard of the event per one unit increase of the metric was determined. A risk classifier was composed from the selected metrics and coefficients. The risk score prediction capacity by LASSO was assessed by means of Harrell’s C-statistic. To assess stratification capacity of the classifier’s derived risk score, a binary risk classifier was generated. Maximally selected rank statistics was used to determine optimal thresholds for defining low- and high-risk groups. Unlike Youden’s index, which was applied in previous studies, this method also incorporates censoring and event timing, allowing for a more relevant prognostic stratification.^27^

Performance of the binary risk classifier was assessed by Kaplan–Meier survival analysis. In addition, hazard ratios (HRs) and confidence intervals (CIs) from a traditional Cox model for association analysis were determined. For validation, the coefficients and thresholds that were derived from the derivation cohort were used to calculate risk scores and its associated risk group in the validation cohort where discriminatory performance was further assessed. The aforementioned analysis steps were performed for primary and secondary endpoints. Furthermore, a time-dependent C-statistic was evaluated across several timepoints and displayed as area under the (receiver operator) curve for accuracy evaluation over time.

For sensitivity analysis, manual metric selection was performed to avoid the abundance of clinically similar metrics and improve face validity of a derived risk score, based on the previous literature and empirical correlations.^12^, ^13^, ^14^ Coefficients were also redetermined by LASSO to assess the predictive and stratification capacity of the manually selected model. As previous publications used traditional Cox stepwise regression analysis, this method was explored for reproducibility purposes in context of highly correlated quantitative MRCP metrics. A *P* value of 0.05 was considered significant. To evaluate whether it would be hypothetically feasible for radiologists to assess the metrics manually, we explored the individual thresholds for prognostic metrics with maximally selected rank statistics.

Statistical analysis was performed with R version 4.3.2. Key packages included *survival* for Cox regression and Kaplan–Meier analysis, *glmnet* for LASSO regression, *gtsummary* and *Hmisc* for descriptive statistics, *survminer* and *ggplot2* for visualisation, and *pROC* & *timeROC* for receiver operating characteristics analysis.

## RESULTS

### Patient Characteristics

**Figure 2:**
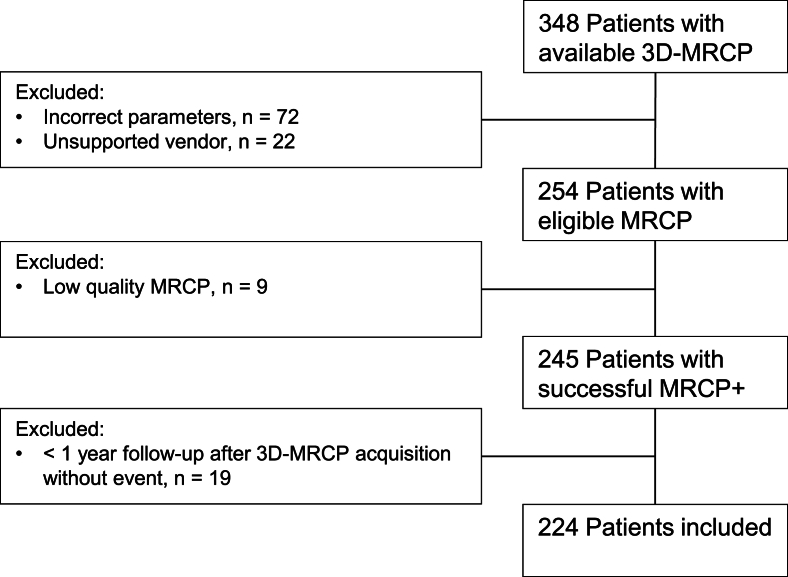
**Flow chart of patient inclusion.** 3D: three-dimensional; MRCP: magnetic resonance cholangiopancreatography.

A total of 224 participants, 200 with large-duct PSC (89%), were included in this study with a 96% success rate of eligible 3D-MRCP to MRCP+ conversions as shown in the flowchart in Figure 2. Not all available 3D-MRCP were suitable for analysis as 29.6% was rejected in pre-analysis because of non-supported parameters or vendor. Patient characteristics are presented in Table 1. Participants from the tertiary centre (Amsterdam UMC) had a significantly lower median time from diagnosis to MRCP+ (3.6 vs 6.4 years) and longer follow-up from MRCP+ to event or censoring (7.8 vs 5.7 years) as compared to transplant centre (Erasmus MC), while total follow-up from diagnosis was similar (12.1 vs 11.1 years). When available, the Amsterdam–Oxford Model risk score showed a nearly significant median higher risk score (1.69 vs 1.57) in the transplantation centre cohort and event rates were also significantly higher (45% vs 27%) when compared to the tertiary centre.

**Table 1:** Patient Demographics at Time of Inclusion.

		Tertiary care centre	Transplant centre	*P value*
		(N = 146)	(N = 78)	
**Sex**	*Male*	90 (61.6%)	53 (67.9%)	*0.43*
**Age at PSC diagnosis (years)**	*Mean (SD)*	36.6 (13.7)	38.4 (14.6)	*0.36*
**Type of PSC**	*Large duct*	125 (85.6%)	75 (96.2%)	*0.03*
	*Small duct*	21 (14.4%)	3 (3.8%)	
**Type of IBD**	*None*	46 (31.5%)	26 (33.3%)	*0.89*
	*UC*	69 (47.3%)	34 (43.6%)	
	*CD*	24 (16.4%)	13 (16.7%)	
	*IBDu*	7 (4.8%)	5 (6.4%)	
**PSC with AIH features**	*Yes*	14 (9.6%)	16 (20.5%)	*0.04*
	*No*	129 (88.4%)	61 (78.2%)	
	*Unknown*	3 (2.1%)	1 (1.3%)	
**Cholecystectomy**	*Performed*	50 (34.2%)	22 (28.2%)	*0.96*
	*Cholecystitis*	8 (16%)	2 (9.1%)	
	*Lithiasis*	22 (44%)	9 (40.9%)	
	*Polyps or GBC*	12 (24%)	9 (40.9%)	
	*Unknown*	8 (16%)	2 (9.1%)	
**History of UDCA use**	*Yes*	105 (71.9%)	74 (94.9%)	*<0.001*
	*No*	24 (16.4%)	0 (0%)	
	*Unknown*	17 (11.6%)	4 (5.1%)	
**Age at MRCP+ (years)**	*Mean (SD)*	43.2 (14.3)	46.2 (14.8)	*0.10*
**Years from PSC diagnosis to MRCP+**	*Median [Q1, Q3]*	3.6 [0.0, 10.3]	6.4 [0.6, 11.8]	*0.03*
**Time from MRCP + to event or censoring (years)**	*Median [Q1, Q3]*	7.8 [5.3, 10.1]	5.7 [2.8, 7.3]	*<0.001*
**Time from diagnosis of PSC to last follow-up (years)**	*Median [Q1, Q3]*	12.1 [8.4, 17.9]	11.1 [6.9, 17.7]	*0.33*
**AOM risk score (n = 152)**	*Median [Q1, Q3]*	1.57 [1.19, 1.97]	1.69 [1.31, 2.53]	*0.06*
**AOM risk category (n = 152)**	*Low*	64 (75.3%)	29 (59.6%)	*0.011*
**Event**	***Liver transplantation or PSC-related death without CRC***	39 (26.7%)	34 (44.8%)	*0.02*
	*Liver transplantation*	25 (17.1%)	21 (26.9%)	
	*Death*			
	*PSC-related death without CRC*	14 (9.6%)	13 (16.7%)	
	*PSC-related death with CRC*	15 (10.3%)	14 (17.9%)	
	*Death by all causes*	21 (14.3%)	14 (17.9%)	

PSC: primary sclerosing cholangitis; SD: standard deviation; IBD: inflammatory bowel disease: AIH: autoimmune hepatitis; GBC: gallbladder carcinoma; UDCA: ursodeoxycholic acid; MRCP+: magnetic resonance cholangiopancreatography: AOM: Amsterdam-Oxford model; CRC: colorectal carcinoma. Categorical data compared with a Chi-squared test if expected count is > 5 and Fisher’s exact test if expected count is < 5 categories; Numerical data compared with Kruskal–Wallis.

### Composing the Risk Classifier With LASSO

The derivation set included 112 patients and 39 composite endpoints (LT- or PSC-related deaths without CRC) and set demographics are shown in Supplementary Table S1. LASSO identified the proportion of ducts with a median range of 3–5 mm, total number of candidate strictures, the sum of stricture length, total number of ducts with stricture or dilatation, time from PSC diagnosis and transplantation centre type as prognostic metrics. The median and interquartile range of all quantitative metrics are displayed in Table 2. Subsequently, the coefficients were implemented into a risk classifier of which the formula is shown in Formula 1. The coefficient path plot, lambda shrinkage plot and lambda regularisation parameters plot are shown in Supplementary Figures S1-S3.

**Table 2:** Quantitative Metrics Selected by LASSO in the Derivation Set.

Prognostic quantitative metric	Median (Q1, Q3)
Ducts with a median 3–5 mm range (%)	20.5 (11.9, 27.7)
Total number of strictures (n)	9 (5, 16)
Total number of ducts with stricture or dilatation (n)	17 (11, 30)
Sum of stricture length (mm)	64.9 (39.1, 125.3)

LASSO: Least absolute shrinkage and selection operator; mm: millimetre; q: quartile.

*Formula 1 - Formula of derived binary risk classifier*

Log(H(t∣X))=h0(t)+0.019·Proportionof3−5mmducts(%)+0.024·Totalnumberofstrictures+0.003·Sumofstricturelength(mm)+0.002·Totalnumberofductswithstrictureordilatation+0.018·TimefromPSCdiagnosis(years)+0.51·Centreofinclusion(iftransplantationcentre)

### Prediction and Stratification Capacity

**Figure 3:**
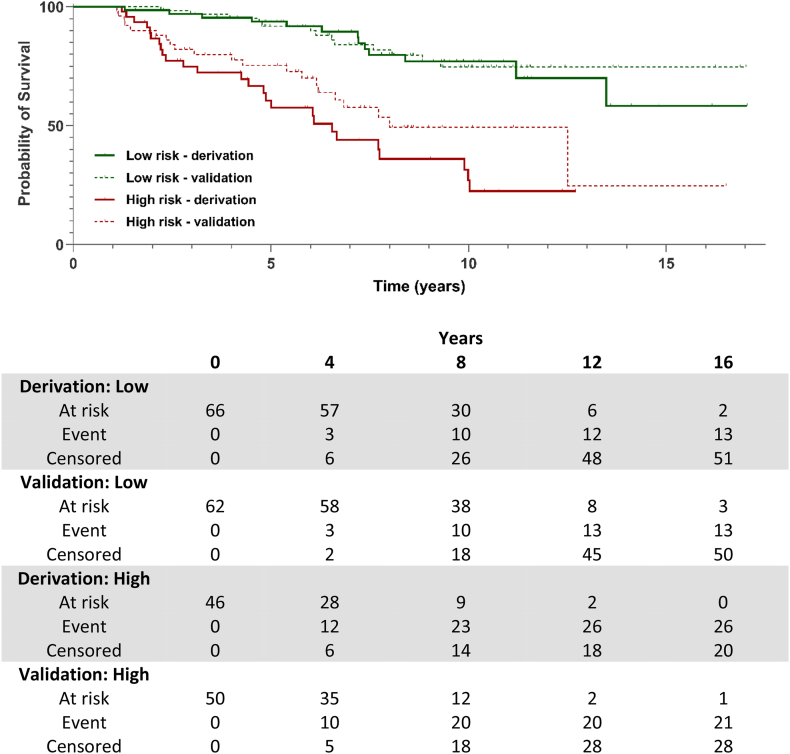
**Transplantation-free survival in PSC patients, stratified in risk groups based on the risk classifier.** Kaplan–Meier survival curve of the high- and low-risk groups in both derivation and validation sets, including the risk table. PSC: primary sclerosing cholangitis.

The median risk score as determined by the risk classifier was 1.08 [0.60–1.45] and the optimal log-relative cut-off of the risk score was 1.14, balancing a sensitivity of 0.67 and specificity of 0.71. The discriminative performance of the risk classifier showed a C-statistic of 0.74 (95% CI: 0.56–0.78). Significant high- versus low-risk stratification capacity was shown by a HR of 5.0 (95% CI: 2.5, 9.9). Prediction analysis in the validation set showed a C-statistic of 0.72 (95% CI: 0.60–0.81) and significant risk group stratification of high versus low-risk with a HR of 3.2 (95% CI: 1.6–6.4). The Kaplan–Meier curve in Figure 3 shows significant differences (*P* ≤ 0.001) in risk group stratification in both derivation and validation set.

**Figure 4:**
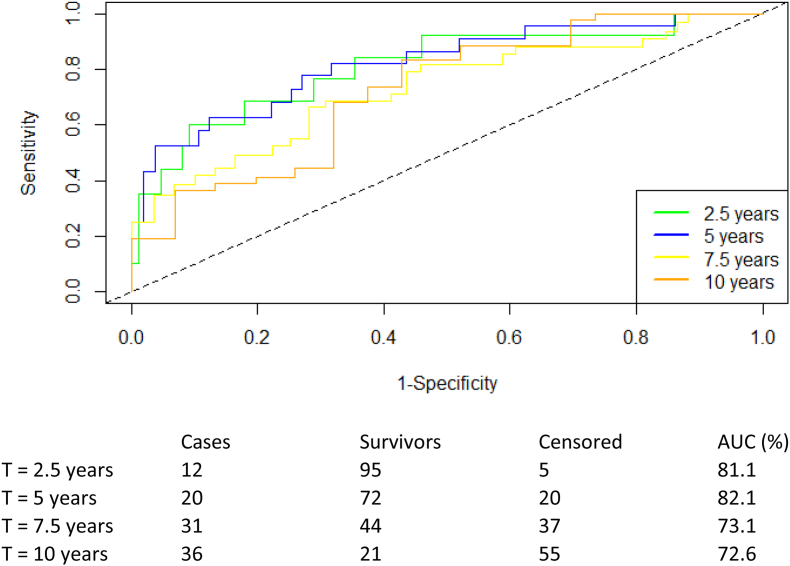
**Time-dependent ROC curves of the risk classifier for transplant-free survival of PSC patients at 2.5, 5, 7.5, and 10 years.** The ROC curves illustrate the classifiers’ discrimination at different timepoints after the MRCP, including a risk table. The diagonal dashed line represents no discriminative ability (C = 0.5). ROC: receiver operating characteristics; PSC: primary sclerosing cholangitis; AUC: area under the curve.

Stratifying by both centre types, the discriminative performances of the classifier in the tertiary care and transplantation centre type were 0.68 (95% CI: 0.53–0.76) and 0.70 (95% CI: 0.55–0.81), respectively.

### Accuracy Assessment

The accuracy assessment showed consistent performance after 2.5 years (81%), 5 years (82%), 7.5 years (73%) and 10 years (73%) as displayed in Figure 4. A decrease in accuracy was seen after year 10 as shown in Supplementary Figure S4, with accuracy dropping to 62% at year 12.5.

### Secondary Endpoints

The composite endpoint of LT- and PSC-related death including death by CRC included 40 events in the derivation set. LASSO identified similar metrics and associated coefficients, with exception of total numbers of ducts with a stricture or dilatation. Nearly identical C-statistics and HR's were found in prediction and stratification assessment, as shown in Supplementary Tables S2 and S3.

For LT and all-cause mortality, 44 events were seen in the derivation set and identical metrics were identified with exception of sum of the stricture length. A minimal decrease in discriminative (C-statistic of 0.72; 0.70 for derivation and validation sets, respectively) performance and stratification capacity was described in both sets as shown in Supplementary Table S3.

### Manual Selection

High correlations were found between total number of candidate strictures and the sum of stricture length (Pearson's Rho, 0.96) and number of ducts with strictures or dilatation (Pearson’s Rho, 0.95) as shown in Supplementary Figure S5. Excluding these from the classifier, the manually composed risk classifier as shown in Formula 2 had a C-statistic of 0.74 (95% CI: 0.56–0.78) in the derivation set and showed a HR of 5.1 (95% CI: 2.6, 10.1, *P* < 0.001) in risk group stratification. In the validation set, a C-statistic of 0.72 (95% CI: 0.60–0.79) was found with significant risk group stratification (HR = 3.4, 95% CI: 1.7, 6.9; *P* < 0.001).

*Formula 2 – Formula of manual risk score*

H(t∣X)=h0(t)+0.020·Proportionof3−5mmducts(%)+0.031·Totalnumberofstrictures+0.018·TimefromPSCdiagnosis(years)+0.521·Centreofinclusion(iftransplantationcentre)

Similar results were found by traditional Cox regression analysis with slightly less discriminative and stratification performance as shown in Supplementary Table 4. Contrary to the manual risk classifier, Cox regression analysis excluded transplant time from PSC diagnosis.

### MRCP+ Metric Thresholds

An optimal cut-off of 28 strictures was found, stratifying patients into low-risk (≤28) and high-risk (>28) groups. High-risk patients had significantly higher hazard rates in comparison with low-risk patients (HR: 4.2; 95% CI: 2.3, 7.6; *P* < 0.001) as visualised in Supplementary Figure S6. An optimal cut-off of 24.3% was found for proportion of 3–5 mm ducts. Patients with a high risk (>24.3%) had higher hazard rates in comparison to low-risk patients (≤24.3%) with a 3.3 HR (95% CI: 2.1, 5.3; *P* < 0.001) as visualised in Supplementary Figure S7.

## DISCUSSION

In this study, we were able to validate the prognostic capacity of quantitative MRCP metrics in the prediction of PSC-related transplantation-free survival and prove its stable accuracy over a 10-year period. Furthermore, a significant risk stratification performance was shown, indicating a potential use of quantitative MRCP metrics as a risk assessment tool in clinical settings.

With our cohort, representing the largest number of subjects to date with the longest follow-up time to assess quantitative MRCP, we identified the total number of strictures and proportion of 3–5 mm ducts as most relevant prognostic metrics. Previous publications also showed the prognostic potential of these metrics with C-statistics ranging from 0.78 to 0.86, strengthening our results and the robustness of these MRCP+ metrics as required by ESGAR as external confirmation is essential for new biomarkers.^12^, ^13^, ^14^ The rationale of these metrics is also in line with ERCP- and MRCP-based risk scores as they evaluate the presence of strictures and dilatations in both extrahepatic and intrahepatic bile ducts in terms of disease severity. The proportion of ducts with a 3–5 mm diameter is likely to relate with the rationale of upstream dilatation features in the DiStrict score and intrahepatic dilatation feature in the ANALI score. Intrahepatic ducts are normally deemed to have a <3 mm diameter while progressive PSC leads to the characteristic beaded pattern and an increase in proportion of ducts with a 3–5 mm diameter might reflect this progression.^28^, ^29^, ^30^ As both qualitative and (semi) quantitative features might share the same rationale, it is likely that quantification could be useful to overcome the subjective nature of current imaging scores.^11^^,^^31^^,^^32^

The need for automated quantification is also emphasised by the challenge that manually counting or calculating strictures or dilatation proportions might introduce in routine clinical practice. For example, we showed that the strongest risk stratification is determined at > 28 for the total number strictures and >24.3% for a proportion of 3–5 mm ducts, which approaches the 26% cut-off described by Cazzagon *et al.*^13^ Manually counting and calculating these features is deemed rather time-consuming and would probably suffer from interobserver variability, limiting the generalisability of these scores if done by hand. In addition, in areas where PSC is extremely rare and expertise is not readily available, automated quantification could provide a great advantage in terms of prognostication or guidance. The size of our cohort allowing a separate validation set and the heterogenous cohort including both transplant and non-transplant centres further supports the generalisable use of quantitative metrics in a risk model, especially since significant differences were found between the tertiary care and transplantation centre while performance is similar when tested on both centre types. This provides additional strength to this study as it is currently the only cohort including tertiary care centre patients.

While this cohort is the largest with the longest follow-up, the slow progression of PSC limits the number of events, thereby restricting the number of prognostic variables that can be included without compromising statistical power. Unlike earlier publications, we used LASSO for metric selection as it is known for its ability to penalise highly correlated metrics, thereby improving model stability and generalisability.^24^, ^25^, ^26^ Furthermore, LASSO was considered more appropriate to avoid overfitting in the context of the limited number of events in relation to the number of (quantitative) covariates. This also drove the decision not to implement further laboratory covariates in current analysis, which requires larger cohorts to remain statistically adequate. Comparisons with cholestatic biomarkers and biomarker-based risk scores were therefore not performed in this study. Although LASSO was deemed the preferred analysis, Cox stepwise regression yielded identical metrics and similar coefficients without inflating predictive performance, underlining the prognostic potential and robustness of quantitative metrics.

In addition, LASSO identified stricture length sum and number of ducts with strictures or dilatation as prognostic quantitative MRCP metrics. However, the manually selected model did not show a relevant change in prediction and stratification capacity, indicating a minimal additional value. In clinical practice, less metrics are preferred to increase face validity and to avoid extensive models that require manual documentation of all features despite the chance of slightly less predictive performance.

Inherently, the retrospective design is a limitation and we acknowledge potential selection bias and the bias of missing data. However, the population-based aspect of the Dutch EpiPSC2 cohort indicates a heterogeneous and representative population, whereas the cohort itself is well-maintained since 2008 and has little missing values of interest for the primary objective of this study. Furthermore, it should be noted that imaging was likely often performed on demand in response to signs of biliary obstruction. This may have facilitated an overestimation by measuring disease exacerbation instead of a stable disease as ERC intervention was not yet performed, limiting its use in stable disease assessment. However, it is yet unknown how quantitative MRCP metrics differ between pre- and post-intervention. Unfortunately, radiological risk scores were not available in this cohort, limiting the ability to directly compare quantitative and qualitative assessment. Likewise, the lack of comparison with cholestatic parameters limits the extent of this study; however, it is commonly accepted that cholestatic biomarkers are impeded by fluctuations or only predictive in late disease stages. Furthermore, comparison with combinations of biochemical markers would introduce significant loss of data as a proportion of the scans used in analysis have no close laboratory assessment available. Therefore, future studies should assess the combination of multiple domains, including quantitative MRCP metrics, laboratory values and clinical information such as disease phenotype as it is likely that this will result in better prognostication.

In conclusion, this study validated the total number of candidate strictures and the proportion of 3–5 mm ducts as prognostic quantitative MRCP metrics. Therefore, future research on their contribution to current radiological and clinical prognostic scores is warranted.

## Credit authorship contribution statement

**Tim E. Middelburg:** Conceptualization; Methodology; Formal Analysis; Investigation; Data Curation; Writing – original draft; Visualization; Project Administration. **Bregje Mol:** Investigation; Data Curation. **Carlos Ferreira:** Software**. Tom Davis:** Methodology**. Karin Horsthuis:** Supervision; Writing – Original Draft. **Ynte S. de Boer:** Supervision; Writing – Original Draft. **Adriaan J. van der Meer:** Data Curation. **Annemarie C. de Vries:** Data Curation. **Roy S. Dwarkasing:** Data Curation. **Johannes A. Bogaards:** Formal Analysis; Methodology; Writing – Original Draft. **Aart J. Nederveen:** Data Curation. **Jaap Stoker:** Supervision; Writing – original draft. **Cyriel Y. Ponsioen:** Conceptualization; Supervision; Writing – Original Draft; Funding acquisition.

## Funding

This study was supported with a grant of Perspectum Ltd but Perspectum Ltd had no further influence on data collection, analysis, reporting, preparation of submission and submission itself.

## Declaration of competing interest

The authors of this manuscript declare relationships with the following companies:

- Carlos Ferreira and Tom Davis are employees of Perspectum Ltd.
- Prof. Dr. Cyriel Y Ponsioen has received funding by Perspectum Ltd. in context of a collaboration agreement between Perspectum Ltd. and the Amsterdam UMC.
