# Supplementary material for: Validation of Quantitative Magnetic Resonance Cholangiopancreatography Metrics in Prediction of Transplant-free Survival in Primary Sclerosing Cholangitis
Source: J Clin Exp Hepatol. 2025 Nov 21;16(1):103417. doi: 10.1016/j.jceh.2025.103417 (PMC12765437; doi:10.1016/j.jceh.2025.103417)
Supplement: Multimedia component 1 [file mmc1.docx]

# Supplementary

Table S1

|  |  | **Derivation set** | **Validation set** |  |
| --- | --- | --- | --- | --- |
| **Variable** |  | **(N=112)** | **(N=112)** |  |
| **Sex** | *Male* | 69 (61.6%) | 74 (66.1%) |  |
| **Age at PSC diagnosis (years)** | *Mean (SD)* | 36.3 (14.1) | 38.3 (13.5) |  |
| **Type of PSC** | *Large Duct* | 100 (89.3%) | 100 (89.3%) |  |
|  | *Small Duct* | 12 (10.7%) | 12 (10.7%) |  |
| **Type of IBD** | None | 35 (31.3%) | 37 (33.0%) |  |
|  | UC | 52 (46.4%) | 52 (46.4%) |  |
|  | CD | 21 (18.8%) | 15 (13.4%) |  |
|  | IBDu | 4 (3.6%) | 8 (7.1%) |  |
| **PSC with AIH features** | *Yes* | 16 (14.3%) | 14 (12.5%) |  |
|  | *No* | 96 (85.7%) | 95 (84.8%) |  |
|  | *Unknown* | 0 (0%) | 3 (2.7%) |  |
| **Cholecystectomy** | *Yes* | 38 (33.9%) | 35 (31.3%) |  |
| **History of UDCA use** | *Yes* | 86 (76.8%) | 93 (83.0%) |  |
|  | *No* | 13 (11.6%) | 11 (9.8%) |  |
|  | *Unknown* | 13 (11.6%) | 8 (7.1%) |  |
| **Age at MRCP+ (years)** | *Mean (SD)* | 43.2 (14.8) | 45.3 (14.3) |  |
| **Years from PSC diagnosis to MRCP+** | *Median [Q1, Q3]* | 5.8 [0.1, 9.3] | 4.0 [0.0, 10.8] |  |
| **Time from MRCP+ to event or censoring (years)** | *Median [Q1, Q3]* | 6.1 [4.2, 9.7] | 7.2 [4.9, 10.0] | |
| **Time from diagnosis of PSC to last follow-up (years)** | *Median [Q1, Q3]* | 11.8 [7.8, 17.6] | 11.8 [7.8, 18.5] |  |
| **AOM risk score (n=132)** | *Median [Q1, Q3]* | 1.66 [1.29, 2.37] | 1.59 [1.16, 2.12] |  |
| **AOM risk category (n=132)** | *Low* | 43 (67.6%) | 57 (82.6%) |  |
| **PSC-related death (without CRC) or liver transplantation** |  | 48 | 34 |  |
| *PSC: Primary sclerosing cholangitis; SD: standard deviation; IBD: Inflammatory bowel disease: AIH: Auto-immune hepatitis; UDCA: ursodeoxycholic acid; MRCP: magnetic resonance cholangiopancreatography: AOM: Amsterdam-Oxford Model; CRC: Colorectal carcinoma* | | | |  |

Figure S1


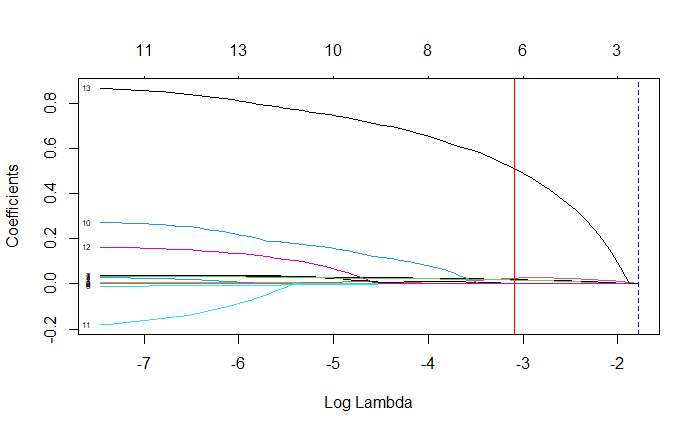


Figure S2
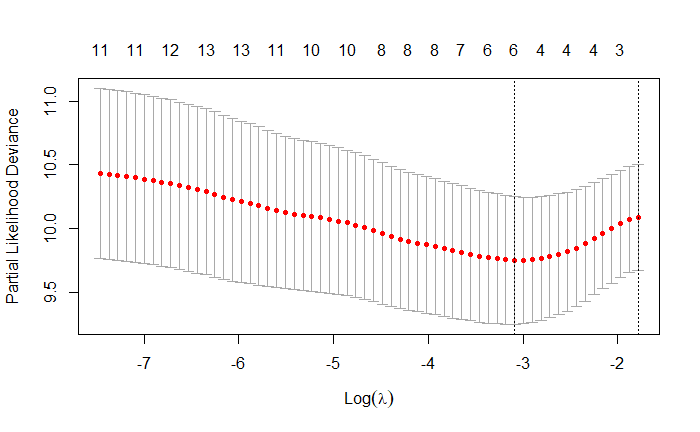


Figure S3
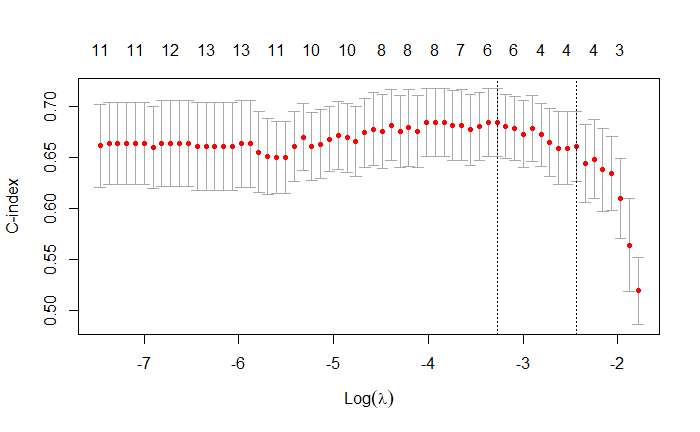


Figure S4

| 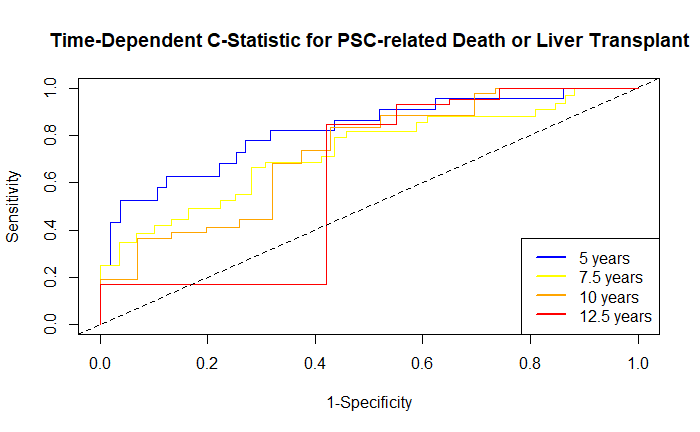 | | | | |
| --- | --- | --- | --- | --- |
|  | Cases | Survivors | Censored | AUC (%) |
| T = 5 | 20 | 72 | 20 | 82.1 |
| T = 7.5 | 31 | 44 | 37 | 73.1 |
| T = 10 | 36 | 21 | 55 | 72.6 |
| T = 12.5 | 38 | 7 | 67 | 61.9 |

Table S2

|  | **PSC-related mortality including CRC and LT** | **All-cause mortality and LT** |
| --- | --- | --- |
| **Variable selected by LASSO** | **Coefficient** | **Coefficient** |
| Proportion of ducts with a median range of 3-5mm (%) | 0.017 | 0.018 |
| Total number of candidate strictures (n) | 0.024 | 0.027 |
| Sum of stricture length | 0.001 | - |
| Total number of ducts with stricture or dilatation | - | 0.005 |
| Time of diagnosis to MRCP+ | 0.013 | 0.018 |
| Centre of inclusion (transplantation) | 0.558 | 0.328 |
| *LASSO: Least Absolute Shrinkage and Selection Operator; PSC: Primary sclerosing Cholangitis; CRC: Colorectal Cancer; LT: liver transplantation: MRCP: Magnetic Resonance Cholangiopancreatography.* | | |

Table S3

| **PSC-related mortality including CRC and LT** | | | | | |
| --- | --- | --- | --- | --- | --- |
| **Set** | **C-statistic** (95% CI) | **Optimal risk score cut-off** | **Sensitivity and specificity** | **HR (95% CI) of high vs low risk group** | **p-value** |
| Derivation | 0.74 (0.56-0.78) | 1.15 | 0.62 and 0.74 | 4.7 (2.4, 9.2) | < 0.001 |
| Validation | 0.72 (0.59-0.82) |  | - | 3.1 (1.6, 6.2) | 0.001 |
| **All-cause mortality and LT** | | | | | |
| **Set** | **C-statistic** (95% CI) | **Optimal risk score cut-off** | **Sensitivity and specificity** | **HR (95% CI) of high vs low risk group** | **p-value** |
| Derivation | 0.72 (0.58-0.80) | 1.42 | 0.40 and 0.91 | 5.3 (2.8, 9.7) | < 0.001 |
| Validation | 0.70 (0.58-0.79) |  | *-* | 3.6 (1.8, 6.8) | < 0.001 |
| *PSC: Primary sclerosing Cholangitis; CRC: Colorectal Cancer; LT: liver transplantation: C-statistic: Conccordance-statistic; CI: confidence intervals; HR: Hazard rate* | | | | | |

Table S4

| **Variables selected by Cox regression for risk classifier** | | | | **HR** | **Coefficient** | | **95% CI** | **p-value** |
| --- | --- | --- | --- | --- | --- | --- | --- | --- |
| Proportion of ducts with a median range of 3-5mm (%) | | | | 1.03 | 0.03 | | 1.00, 1.07 | 0.056 |
| Total number of candidate strictures (n) | | | | 1.04 | 0.04 | | 1.01, 1.07 | 0.002 |
| Centre of inclusion (transplantation) | | | | 2.06 | 0.72 | | 1.17, 4.28 | 0.032 |
| **Performance of classifier for composite of LT and PSC-related death excluding CRC** | | | | | | | | |
| **Set** | **C-statistic** (95% CI) | **Optimal risk score cut-off** | **Sensitivity and specificity** | | | **HR (95% CI) of high vs low risk group** | | **p-value** |
| Derivation | 0.72 (0.54-0.77) | 2.31 | 0.58, 0.74 | | | 4.3 (2.2, 8.3) | | < 0.001 |
| Validation | 0.70 (0.61-0.79) |  | *-* | | | 2.8 (1.4, 5.6) | | 0.003 |
| *HR: Hazard Rate; CI: confidence intervals; LT: Liver transplantation: PSC: PSC: Primary sclerosing Cholangitis; CRC: Colorectal carcinoma; C-statistic: Conccordance-statistic.* | | | | | | | | |

Figure S5


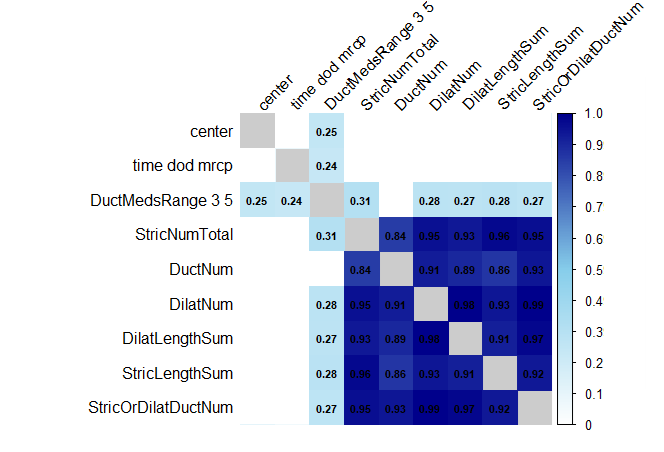


Figure S6

|  | **Years** | **0** | **5** | **10** | **15** |
| --- | --- | --- | --- | --- | --- |
| **Low risk** | *At risk* | 201 | 146 | 49 | 6 |
|  | *Event* | 0 | 27 | 55 | 59 |
| **High risk** | *At risk* | 23 | 9 | 0 | 0 |
|  | *Event* | 0 | 10 | 14 | 14 |

Figure S7

|  | **Years** | **0** | **5** | **10** | **15** |
| --- | --- | --- | --- | --- | --- |
| **Low risk** | At risk | 146 | 116 | 41 | 5 |
|  | Event | 0 | 12 | 30 | 34 |
| **High risk** | At risk | 78 | 39 | 8 | 1 |
|  | Event | 0 | 25 | 39 | 39 |
